# Electrochemical Ultrasensitive Sensing of Uric Acid on Non-Enzymatic Porous Cobalt Oxide Nanosheets-Based Sensor

**DOI:** 10.3390/bios12121140

**Published:** 2022-12-07

**Authors:** Sakeena Masrat, Vandana Nagal, Marya Khan, Iqra Moid, Shamshad Alam, Kiesar Sideeq Bhat, Ajit Khosla, Rafiq Ahmad

**Affiliations:** 1 Sensors Lab, Centre for Nanoscience and Nanotechnology, Jamia Millia Islamia, New Delhi 110025, India; 2 Quantum and Nanophotonics Research Laboratory, Centre for Nanoscience and Nanotechnology, Jamia Millia Islamia, New Delhi 110025, India; 3 Department of Life Science, Shakuntala Memorial Educational Institute, Bahraich 271870, India; 4 Department of Pharmacology & Therapeutics, Rosewell Park Cancer Institute, Elm Street & Carlton Street, Buffalo, NY 14263, USA; 5 Department of Bioresources, University of Kashmir, Hazratbal, Srinagar 190006, India; 6 Singapore-MIT Alliance for Research and Technology (SMART), Critical Analytics for Manufacturing Personalized-Medicine (CAMP), Create Way 138602, Singapore; 7 Department of Applied Chemistry, School of Advanced Materials and Nanotechnology, Xidian University, Xi’an 710126, China

**Keywords:** cobalt oxide, porous, nanosheets, uric acid, electrochemical, non-enzymatic, ultra-sensitive, sensor

## Abstract

Transition metal oxide (TMO)-based nanomaterials are effectively utilized to fabricate clinically useful ultra-sensitive sensors. Different nanostructured nanomaterials of TMO have attracted a lot of interest from researchers for diverse applications. Herein, we utilized a hydrothermal method to develop porous nanosheets of cobalt oxide. This synthesis method is simple and low temperature-based. The morphology of the porous nanosheets like cobalt oxide was investigated in detail using FESEM and TEM. The morphological investigation confirmed the successful formation of the porous nanosheet-like nanostructure. The crystal characteristic of porous cobalt oxide nanosheets was evaluated by XRD analysis, which confirmed the crystallinity of as-synthesized cobalt oxide nanosheets. The uric acid sensor fabrication involves the fixing of porous cobalt oxide nanosheets onto the GCE (glassy carbon electrode). The non-enzymatic electrochemical sensing was measured using CV and DPV analysis. The application of DPV technique during electrochemical testing for uric acid resulted in ultra-high sensitivity (3566.5 µAmM^−1^cm^−2^), which is ~7.58 times better than CV-based sensitivity (470.4 µAmM^−1^cm^−2^). Additionally, uric acid sensors were tested for their selectivity and storage ability. The applicability of the uric acid sensors was tested in the serum sample through standard addition and recovery of known uric acid concentration. This ultrasensitive nature of porous cobalt oxide nanosheets could be utilized to realize the sensing of other biomolecules.

## 1. Introduction

Uric acid (UA) plays an important role in various biological processes and physiological functions in humans and higher species [1]. Bodily fluids (i.e., serum, urine, and saliva) contain UA. Mainly, the liver and intestines are the primary sites of UA production. However, the majority of UA is removed by urate transporters in the kidneys and intestines. Unusually low, excessive, or variable UA concentration is an indicator of various diseases (i.e., pneumonia, gout, leukemia, type 2 diabetes, chronic renal diseases, toxemia during pregnancy, multiple sclerosis, metabolic disorders, and hypertension) [2,3,4,5]. Therefore, monitoring UA concentration becomes crucial for early disease diagnosis since UA acts as a diagnostic marker for UA-concentration associate diseases. 

Several standard analytical methods (i.e., flow-injection analysis, chromatography, HPLC, chemiluminescence, mass spectrometry, colorimetry, capillary electrophoresis-amperometry, and enzyme test kits) are utilized to determine UA concentration [6,7,8,9,10]. However, complex sample preparation, high cost, slow preparation, and skilled technician requirements are the major drawbacks of the above-mentioned analytical methods [11]. On the other hand, electrochemical-based sensors have the potential for rapid detection and offer high-sensing performance during biomolecules detection [8,9,10,12,13,14]. Recently, considerable attention has been given to electrochemical-based UA sensors due to their high selectivity, accuracy, less interference, and low-cost [15,16,17,18,19,20].

There are two types of electrodes (i.e., metal-based and carbon-based electrodes) that are mostly used in electrochemical sensor fabrication. The carbon-based electrodes (i.e., glassy carbon, screen-printed, and carbon-paste electrodes) are the preferred working electrodes due to the minimum over potential requirement compared to the metal-based electrodes [19]. Due to their low costs and ease of preparation, the carbon-based electrodes have been extensively used in electrochemical sensing applications. These conventional bare electrodes are not preferred due to their weak electrochemical oxidation kinetics [20]. Hence, nanomaterial/electrocatalyst (as redox dynamic site) modification is needed to improve the electrochemical oxidation kinetics of the bare electrodes [21]. The choice of nanomaterial/electrocatalyst modifiers (for example, metals/metal oxides or carbon-based nanomaterials) depends on the type of the electrode and analytical requirements.

The improved electro-catalytic activity and charge transfer capabilities of metal/metal oxide have attracted considerable interest for their applications in electrode modifications [15,16,17,18,19,20,21,22,23,24,25,26,27]. Among different metal oxides, cobalt oxide nanomaterials have been utilized to fabricate enzymatic/non-enzymatic sensors where the use of cobalt oxide nanostructures enhances the desired electrochemical properties [23,24,25,26,27,28,29]. Different methods (i.e., thermal decomposition, sol-gel, surfactant-mediated synthesis, spray-pyrolysis, and polymer-matrix assisted) are utilized for the synthesis of various kinds of cobalt oxide nanostructures. Recently, cobalt oxide has attained interest for different applications (i.e., heterogeneous catalysis, lithium-ion batteries, gas sensing, electrochemical sensors, and solar cells). Cobalt oxide nanostructures are the preferred material to modify the electrode, which offers abundant active sites for reaction and easy adsorption/electroactive species diffusion [24]. Hence, cobalt oxide nanostructures modified sensor electrodes are suitable for the detection of different analytes. For example, Nagal et al. described the use of nano berry-like cobalt oxide nanostructures for the electrochemical-based enzyme-less uric acid sensor [24]. Kogularasu et al. investigated the impact of cobalt oxide polyhedrons to develop an enzyme-free biosensor detect H_2_O_2_ [25]. Zhang and Liu utilized cobalt oxide nanosheets for enzyme-free detection of glucose [26]. Kang et al. utilized cobalt oxide nanowires for the fabrication of an enzyme-less glucose sensor [27]. Mondal et al. fabricated glucose sensors using different nanostructures (like spherical nanoparticles, porous nanorods, and nanoflowers) of cobalt oxide [28]. Chang et al. evaluated the electrochemical sensing performance of lactic acid using cobalt oxide nanostructures [29]. Therefore, there is a demand for designing cobalt oxide nanostructures having excellent surface area, which can be utilized to fabricate high-performance sensors.

In this work, we demonstrated the synthesis of porous cobalt oxide nanosheets using a simple and low-temperature-based hydrothermal method. The porous cobalt oxide nanosheets were comprehensively characterized using XRD, FE-SEM, and TEM. The electrochemical properties of porous cobalt oxide nanosheet-based non-enzymatic UA sensors were studied by cyclic voltammetry (CV), EIS (electrochemical-impedance-spectroscopy), and DPV (differential-pulse voltammetry). The porous cobalt oxide nanosheets-based non-enzymatic UA sensor exhibited ultra-high sensitivity (3566.5 µAmM^−1^cm^−2^) when analyzed with the DPV technique. Additionally, selectivity, stability, and applicability in serum samples were evaluated. This porous cobalt oxide nanosheet-based non-enzymatic UA sensor offers better sensitivity when compared to CV-measured sensitivity (470.4 µAmM^−1^cm^−2^).

## 2. Materials and Methods

### 2.1. Chemicals

All analytical chemicals were obtained from Sigma Aldrich and used. Cobalt nitrate hexahydrate (≥99.99%; Co(NO_3_)_2_.6H_2_O), sodium hydroxide pellets (≥97%; NaOH), potassium chloride (KCl), uric acid (≥99%), ethylene glycol (99.8%), sodium chloride (NaCl), potassium hexacyanoferrate [K_3_Fe(CN)_6_]^3−/4−^ (≥99%), glucose, fructose, lactic acid, L-cysteine, urea, phosphate buffer saline (PBS; pH = 7.4) solution, 2-(2-Butoxyethoxy)ethyl acetate (≥99.2%), dopamine, and ascorbic acid were purchased. In our experiments, ultrapure water was utilized.

### 2.2. Porous Cobalt Oxide Nanosheets Synthesis

**Scheme 1:**
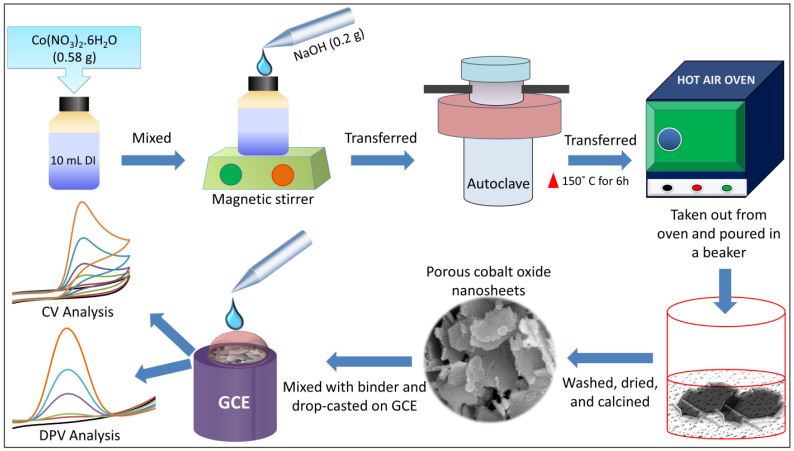
Schematic showing the synthesis process of porous cobalt oxide nanosheets, sensor fabrication process, and measurement techniques used.

A simple low-cost hydrothermal process is used to synthesize porous cobalt oxide nanosheets (Scheme 1). For synthesis, first, a precursor solution of Co(NO_3_)_2_.6H_2_O (0.58 g) was prepared in 10 mL DI water, and then NaOH (0.2 g) solution was dropwise added while vigorous stirring for 30 min to obtain a homogenous mixture. The prepared solution was transferred into an autoclave vessel (Teflon-lined stainless steel) and put into a hot air oven at 150 °C for 6h. On completion of the reaction, the autoclave was cooled down and the powder sample was washed multiple times with DI and ethanol. Finally, the black precipitate was dried (at 60 °C) and annealed (at 500 °C) for 3h before characterizing it in detail.

### 2.3. Sensor Fabrication

The porous cobalt oxide nanosheets-based non-enzymatic UA sensor was fabricated using a conductive binder along with cobalt oxide nanosheets (Scheme 1). In brief, a slurry of 0.01 g porous cobalt oxide nanosheets and 50 µL conductive binder (2-(2-Butoxyethoxy)ethyl acetate) was prepared using mortar and pestle. The slurry was sonicated for 10 min before fixing onto the working electrode surface. Different amounts of slurry were fixed on the working electrode to optimize the most suitable amount of slurry, which gives the best sensing performance (for example, 4 µL was the optimized amount). The porous cobalt oxide nanosheet-based sensor was dried at 60 °C for 6h and kept at room temperature.

### 2.4. Materials Characterization and Electrochemical Analysis of Sensor

The FESEM (field-emission-scanning electron microscope; Zeiss, Sigma) was utilized to analyze porous sheet-like cobalt oxide nanostructures morphology. The structural analysis was examined using XRD (Rigaku), where Cu-Kα X-ray radiation (λ = 1.5418 Å), current (30 mA), and voltage (40 kV) were used. A more detailed study of porous sheet-like cobalt oxide nanostructures was characterized with TEM (TECNAI G20; accelerating voltage = 200 kV). The ASAP 2010 analyzer and Barrett-Joyner-Halenda (BJH) method were utilized for nitrogen adsorption-desorption analysis (at 77 K) and pore size distribution determination, respectively.

For the electrochemical measurements of the fabricated porous cobalt oxide nanosheets-based non-enzymatic UA sensor, a compact and portable potentiostat/impedance analyzer “PalmSens4” was used. The CV and EIS were used to evaluate the best-performing sensor using the optimum amount of porous cobalt oxide nanosheets in a redox probe solution ([Fe(CN)_6_]^3−/4−^). During EIS measurement, frequency range and applied potential were set to 0.01 Hz to 100 kHz and 0.25 V, respectively. The sensing performance of the fabricated porous cobalt oxide nanosheet-based non-enzymatic UA sensor was evaluated using CV and DPV techniques.

## 3. Results

### 3.1. Characterizations of Porous Cobalt Oxide Nanostructures

**Figure 1:**
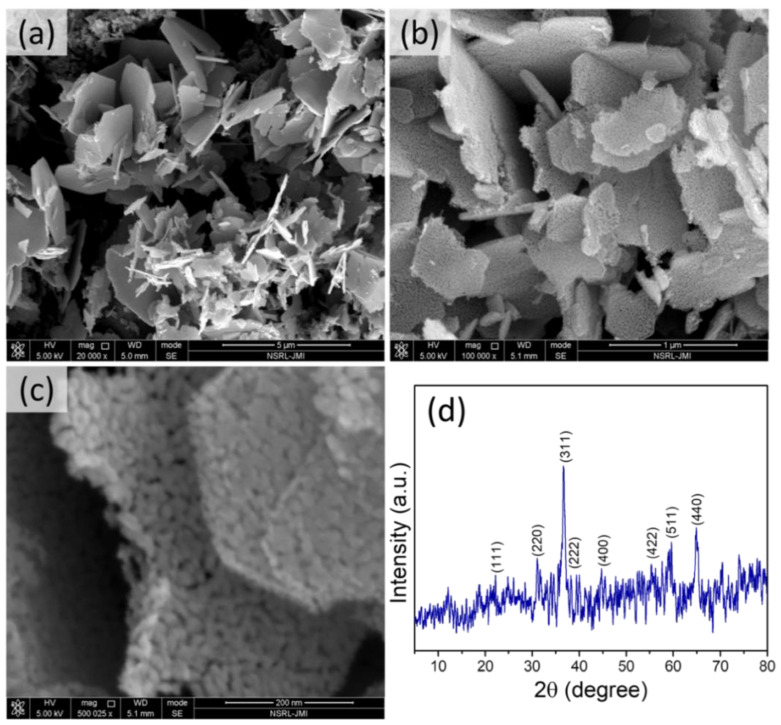
FESEM images at low- (**a**,**b**) and high- (**c**) magnifications and XRD analysis (**d**) of porous cobalt oxide nanostructure.

**Figure 2:**
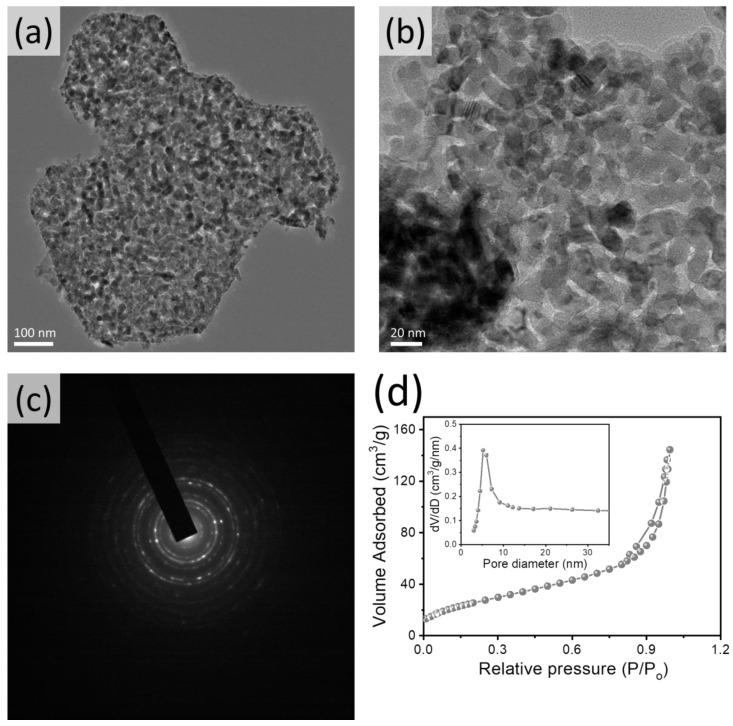
TEM images at low- (**a**), high- (**b**) magnifications, SAED pattern (**c**), and BET Nitrogen adsorption-desorption isotherms of porous cobalt oxide sheet-like nanostructure. Inset (**d**) shows the pore size distribution plot of cobalt oxide sheet-like nanostructure.

The surface morphology and crystallinity of as-synthesized cobalt oxide nanostructure were characterized using FESEM, TEM, and XRD. Figure 1a–c displays the surface morphology of the as-synthesized cobalt oxide nanostructure. The low-magnification images of cobalt oxide nanostructures reveal that the synthesized nanomaterial is obtained in bulk amounts with irregular shapes and sizes (Figure 1a,b). The high-magnification image shows that cobalt oxide nanostructures bear porous sheet-like morphology (Figure 1c). Only the surface morphology is smooth, with uniform pores present on the surface. Figure 2d shows the XRD pattern of as-synthesized cobalt oxide nanomaterial. The XRD pattern confirms the crystalline nature of nanomaterial, and the obtained pattern is well indexed (JCPDS card no. 42-1467) [30]. The diffraction peaks of cobalt oxide are observed at 2Ɵ values of 22.1°, 31.1°, 36.6°, 38.1°, 44.6°, 55.4°, 59.6°, and 66.7°, corresponding to the miller indices of [111], [220], [311], [222], [400], [422], [511], and [440], respectively. The highest intensity diffraction peak is seen along [311] lattice plane. To confirm the porosity, TEM analysis of the porous cobalt oxide sheet-like nanostructure was done (Figure 2). The TEM images show the porous sheet-like nanostructure of cobalt oxide. Additionally, the SAED pattern (Figure 2c) of the porous cobalt oxide sheet-like nanostructure suggests the crystal nature. These observations are supported by FESEM images. The surface area and pore size of the porous cobalt oxide sheet-like nanostructure was analyzed using BET (Brunauer-Emmett-Teller) analysis, shown in Figure 2d. The isotherm pattern indicates the porous nature of nanomaterial. The obtained BET surface area of porous cobalt oxide sheet-like nanostructure is around ~166 m^2^/g. A narrow pore size distribution from 3 to 7 nm was obtained with an average pore size of ~5 nm (Inset of Figure 2d). The high surface area and small pore size of the porous cobalt oxide sheet-like nanostructure will offer a better catalytic for electrochemical reactions.

### 3.2. Electrochemical Studies using CV and EIS Techniques

**Figure 3:**
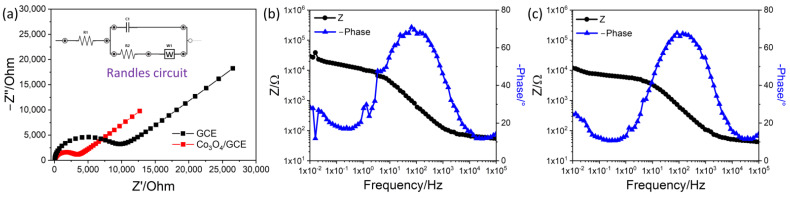
EIS spectra of bare GCE and cobalt oxide/GCE electrodes recorded in the redox probe solution of [Fe(CN)_6_]^3−/4−^ with KCl (0.1 M) “as supporting electrolyte”. (**a**) Nyquist plots of bare GCE (black line) and cobalt oxide/GCE (red line) electrodes and their respective Bode plots (**b**,**c**). Inset a shows the Randle circuit.

The electrochemical properties of bare GCE and cobalt oxide/GCE electrodes were analyzed using EIS and CV techniques. The electron transfer reaction of bare GCE and cobalt oxide/GCE electrodes for the redox probe solution of [Fe(CN)_6_]^3−/4−^ with KCl (0.1 M) determine the kinetic parameters (i.e., electron transfer rate and charge transfer resistance) (Figure 3). The EIS was carried out at 0.25 V in the frequency range of 0.01 Hz to 100 kHz. Nyquist plots in Figure 3a show two frequency regions, one at a higher and another at a lower frequency region. Cobalt oxide/GCE electrode showed a small semicircle (at a higher frequency region) and a straight line (at a lower frequency region), which suggests a perfect diffusion-controlled process during the electron transfer reaction. The bare GCE electrode showed a higher charge transfer resistance (R_ct_) compared to the modified GCE electrode. The bode plots of bare GCE and cobalt oxide/GCE electrodes are illustrated in Figure 3b,c, respectively. It can be seen from these bode plot curves that the modifying GCE with cobalt oxide decreased the interfacial impedance. Additionally, the bode angle was decreased after GCE surface modification with cobalt oxide. The shift of lower frequency peak for cobalt oxide modified GCE suggests the more prominent electron transfer process compared to the bare GCE electrode [31].

**Figure 4:**
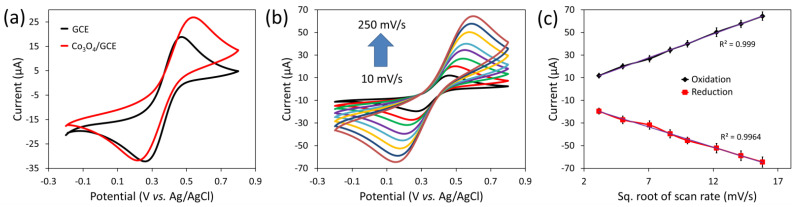
(**a**) CV response curves of bare GCE and cobalt oxide/GCE electrodes recorded in the redox probe solution of [Fe(CN)_6_]^3−/4−^ with KCl (0.1 M) “as supporting electrolyte”. The CV curves were measured at the fixed scan rate (50 mV/s). (**b**) CV analysis of cobalt oxide/GCE electrode at varying scan rates and respective calibration plot (**c**). In figure (**c**), the arrow shows the scan rate increasing direction from 10 mV/s to 250 mV/s.

The CV data obtained for the bare GCE and cobalt oxide/GCE electrodes agree with the EIS data. The CV response curves were recorded from −0.2V to + 0.8V (vs. Ag/AgCl) at a fixed scan rate (50 mV/s) (Figure 4a). Clear redox peaks (i.e., oxidation and reduction peaks) can be seen in the obtained CV response curves, where the cobalt oxide/GCE electrode showed improved oxidation peak current value compared to bare GCE. Additionally, the effect of scan rate on the cobalt oxide/GCE electrode’s electron transfer characteristics was investigated by measuring CV response curves at different scan rates (i.e., 10–250 mV/s) (Figure 4b). It can be seen from the CV curves that the oxidation and reduction peak current values increase with the scan rate increase. The CV curve shape and the values of peak potential separation indicate a diffusion-controlled process in the redox probe solution. To verify the diffusion-controlled process over the cobalt oxide/GCE electrode, a plot of current peak vs. square root of scan rate is plotted in Figure 4c. A perfect linear relationship was observed between the square root of the scan rate and the value of the peak current. This further confirms the diffusion-controlled process over the surface of the modified electrode [32,33,34].

### 3.3. Sensing Performance Characterization using CV Technique

**Figure 5:**
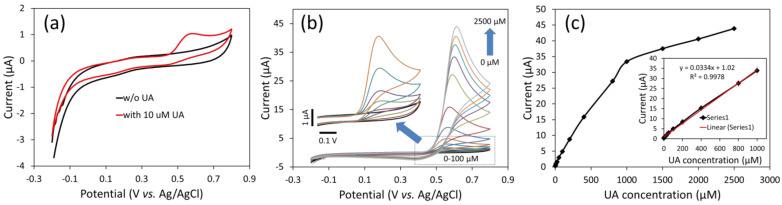
(**a**) CV response curves recorded for cobalt oxide/GCE electrode in PBS without and with uric acid (10 µM) at 50 mV/s, (**b**) CV responses of cobalt oxide/GCE electrode with increasing uric acid concentrations (0−2500 µM), and (**c**) plot of current response vs. uric acid concentration showing linear and non−linear regions. Inset b shows the magnified CV response curves for low-concentration uric acid. Inset c shows the calibration plot of the linear region (i.e., response vs. uric acid concentration).

**Scheme 2:**
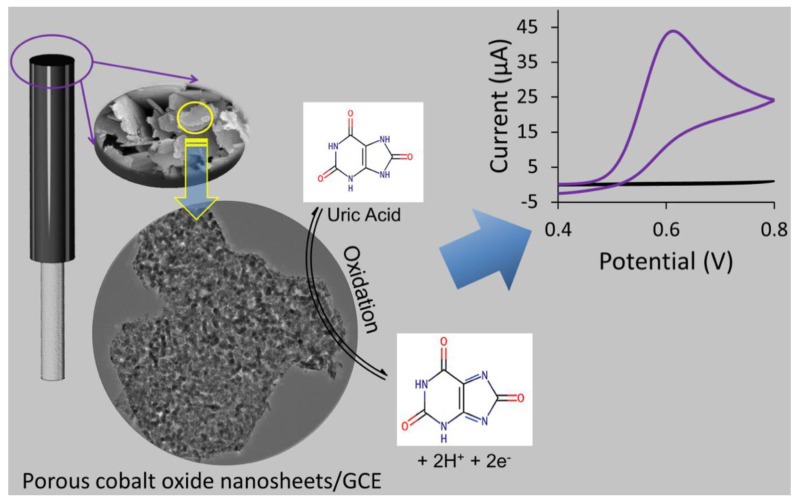
Scheme showing uric acid oxidation over the porous cobalt oxide nanosheets/GCE sensor.

The response of the cobalt oxide/GCE sensor was characterized towards uric acid before a detailed analysis of sensing performance. The CV analysis of the cobalt oxide/GCE sensor was performed in PBS without and with uric acid (10 µM) at 50 mV/s (Figure 5a). When CV analysis was done in PBS, there was no noticeable peak in the CV curve. However, in 10 µM uric acid, a noticeable peak of uric acid oxidation was present at 0.6 voltage. The possible and most accepted detection mechanism for uric acid oxidation involves the transfer of two-electron/two-proton, which enhances the response during sensing measurement (Scheme 2) [35,36,37]. Additionally, the porous nature of nanosheets provided abundant catalytic sites due to the large surface area. Moreover, cobalt oxide is a p-type semiconductor, which could provide excess hole concentration and help to capture the electrons during uric acid oxidation. Also, we observed the irreversible oxidation peak in the CV curve that indicated swift electron transfer between the GCE and porous cobalt oxide during the electrochemical detection of uric acid.

To evaluate the sensing performance (i.e., sensitivity, detection range, and detection limit), the CV response curves of the cobalt oxide/GCE sensor were measured with increasing concentrations of uric acid (0–2500 µM) as shown in Figure 5b. In this figure, the CV curves showed an increase in current with increasing uric acid concentration. A graph of peak current (µA) vs. uric acid concentration (µM) was drawn (Figure 5c). From this graph, two regions (linear and non-linear) can be seen. In general, the non-linear region signifies the saturation of the current response of the cobalt oxide/GCE sensor on those uric acid concentrations (i.e., high uric acid concentration). Further, the linear region of the sensor response is taken and a calibration plot (peak current (µA) vs. uric acid concentration (µM) is plotted, shown in the inset of Figure 4c. The sensor responded linearly up to 1000 µM of uric concentration (regression coefficient (R^2^) = 0.9978). From the slope, we calculated the sensitivity of 470.4 µAmM^−1^cm^−2^ [38]. Additionally, based on the S/N ratio = 3, the detection limit was calculated to be 10 µM. The obtained sensitivity, linear range, and detection limit were comparatively better than most of the previously reported literature (Table 1). The good sensing performance is due to high surface-to-volume ratio and the presence of large active sites on as-synthesised porous nanosheets like nanomaterial.

### 3.4. Sensing Performance Characterization using DPV Technique

**Figure 6:**
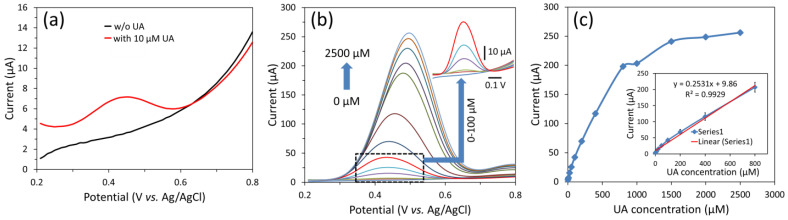
(**a**) DPV response curves recorded for cobalt oxide/GCE sensor in PBS without and with uric acid (10 µM), (**b**) DPV responses of cobalt oxide/GCE electrode with increasing uric acid concentrations (0–2500 µM), and (**c**) plot of current response vs. uric acid concentration showing linear and non-linear regions. Inset b shows the magnified DPV response curves for low-concentration uric acid. Inset c shows the calibration plot of the linear region (i.e., response vs. uric acid concentration).

The DPV technique is more sensitive than CV due to the minimization of capacitive current. For this reason, we utilized the DPV technique to evaluate the sensing performance of the cobalt oxide/GCE sensor. Initially, the electrochemical behavior of the cobalt oxide/GCE sensor was measured. Figure 6a shows DPV curves obtained in PBS buffer (pH 7.4) without and with 10 µM uric acid. The appearance of the uric acid oxidation peak at 0.45 potential (vs. Ag/AgCl), compared to the DPV curve recorded in PBS buffer solution, indicated the sensitivity nature of the cobalt oxide/GCE sensor towards uric acid. Additionally, when measuring CV response (as shown in Figure 5a), it was seen that uric acid oxidation is an irreversible process.

Then, DPV was performed with increasing uric acid concentration (up to 2500 µM) in PBS buffer. The obtained DPV curves are shown in Figure 6b, where an increase in current can be seen with increased uric acid concentration. A plot of peak current (µA) vs. uric acid concentration (µM) is shown in Figure 6c along with the calibrated plot of the linear range of the sensor in the inset. The cobalt oxide/GCE sensor showed a linear range of up to 800 µM uric acid concentration with R^2^ of 0.9929. However, the current level was decreased at a higher uric acid concentration due to the saturation of electrocatalysis of uric acid on the electrode surface. The sensitivity of the cobalt oxide/GCE sensor was calculated by using the standard equation of slope of the calibrated plot/working electrode surface area. The sensor showed the highest sensitivity of 3566.5 µAmM^−1^cm^−2^. The limit of detection was 12 µM. The achieved sensing performance results are shown in Table 1. As shown in Table 1, the cobalt oxide/GCE sensor showed ultra-high sensitivity compared to previously reported literature [11,24,30,39,40,41,42,43,44,45,46]. Furthermore, the DPV technique showed ~7.58 times high sensitivity (3566.5 µAmM^−1^cm^−2^) compared to CV’s measured sensitivity (470.4 µAmM^−1^cm^−2^). These results confirmed the fact that DPV is a more sensitive technique as compared to CV. However, the main reason for getting high sensing performance is attributed to large active sites and the surface-to-volume ratio of as-synthesised porous nanosheets like nanomaterial.

### 3.5. Interference and Stability Tests of Cobalt Oxide/GCE Sensor

**Figure 7:**
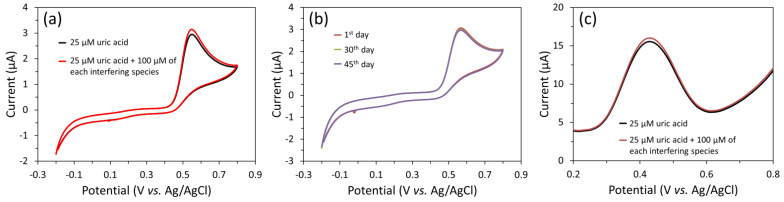
(**a**) CV response curves recorded for cobalt oxide/GCE sensor in PBS with 25 µM uric acid only (black curve) and 25 µM uric acid and 100 µM of each interfering species (i.e., lactic acid, L−cysteine, glucose, urea, fructose, sodium chloride, and potassium chloride) (red curve). (**b**) CV response curves of cobalt oxide/GCE sensor for 25 µM uric acid showing stability of sensor after 30 and 45 days. (**c**) DPV response curves for 25 µM uric acid only (black curve) and 25 µM uric acid and 100 µM of each interfering species (i.e., ascorbic acid, dopamine, and urea).

We investigated the selectivity of the cobalt oxide/GCE sensor for uric acid detection in the presence of possible interferences. Eight possible interfering species, including lactic acid, L-cysteine, glucose, urea, fructose, sodium chloride, and potassium chloride, were taken for the selectivity study. Figure 7a illustrates CV curves for 25 µM uric acid only and 25 µM uric acid and 100 µM of each interfering species (i.e., lactic acid, L-cysteine, glucose, urea, fructose, sodium chloride, and potassium chloride). There is a slight increase (positive interference) in CV response with a high concentration of interfering species. Based on this result, the cobalt oxide/GCE sensor is selective for uric acid determination. Additionally, we evaluated the sensor stability after storing sensor at room temperature and measuring the response after 30 and 45 days for 25 µM uric acid (Figure 7b). As shown in Figure 7b, the sensor showed good stability and maintained 97.4% of its current peak after 45 days of storage. Additionally, the low RSD of 2.6% indicated good stability. Finally, we tested selectivity tests in the presence of ascorbic acid, dopamine, and urea (Figure 7c). In the presence of these species, a slight increase in current response was noticed. However, no other peaks were noticed.

### 3.6. Analysis of Real Serum Sample

To determine whether the cobalt oxide/GCE sensor was suitable for uric acid detection in human serum samples (obtained from Sigma−Aldrich; H4522). We used a standard addition- (known uric acid concentration) based method to estimate the recovery results of the added uric acid concentration in a serum sample. The recovery (%) was calculated using formula [Recovery (%) = Calculated uric acid concentration × 100/Added uric acid concentration]. The obtained data are shown in Table 2. Recovery results showed that the cobalt oxide/GCE sensor was suitable for uric acid determination in the real sample.

## 4. Conclusions

In this study, a low temperature-based hydrothermal method was utilized to synthesize porous nanosheets-like cobalt oxide nanostructures. The crystallinity and morphology of as-synthesized cobalt oxide nanostructures were tested using direct techniques (i.e., FESET, TEM, and XRD). The obtained results showed the successful formation of the porous nanosheet-like nanostructure that bears good crystallinity. The possibility of using such porous nanosheets-like cobalt oxide nanostructures in the sensor was tested by using electrochemical methods, such as CV and EIS. Based on the obtained data, sensing performance evaluation using CV and DPV techniques indicated high sensitivity. The DPV is a more sensitive technique as compared to CV. In this context, DVP data showed ultra-high sensitivity (3566.5 µAmM^−1^cm^−2^), which was ~7.58 times better than CV-based sensitivity (470.4 µAmM^−1^cm^−2^). Additionally, the cobalt oxide/GCE sensor exhibited good selectivity during uric acid measuring in the interfering species. The stability and applicability of the cobalt oxide/GCE sensor were tested, which showed good stability and applicability in a serum sample. Nevertheless, this work contributes to obtaining ultra-high sensitivity using porous cobalt oxide nanosheet-like nanostructures and provides the further possibility to improve sensing performance with surface modification of nanosheets using other metal/metal oxides.

## Figures and Tables

**Table 1:** Sensing performance comparison with previous reports based on electrochemical studies.

Modified Electrode	Method of Detection	Sensitivity (µAcm^−2^mM^−1^)	Linear range (µM)	Detection limit (µM)	Ref.
g-C_3_N_4_ NSs/GCE	DPV	-	100–1000	4.5	[11]
Co_3_O_4_ nanoberries/GCE	CV	206	5–3000	~2.4	[24]
Co_3_O_4_ nanostructures/GCE	CV	-	500–530	100	[30]
Ag-Fe_2_O_3_@PANI	DPV	128.29	0.001–0.900	0.000102	[39]
Nafion/Uricase/ZnO/Au	Amperometry	89.74	100–590	25.6	[40]
Cu_2_O/ferrocene/uricase/GCE	DPV	1.9	0.1–1000	0.0596	[41]
Fe_3_O_4_@SiO_2_/MWCNT	SWV	0.303	0.60–100	0.13	[42]
Uricase/PPD/PrB-SPCE	CV	4.9	50–1000	18.7	[43]
ITO-rGO-AuNPs	LSV	0.31	10–500	3.6	[44]
PrGO/PB 100/GCE	CV	-	40–415	8.0	[45]
ZIF-11/GCE	DPV	174	20–540	0.48	[46]
Co_3_O_4_ porous NSs/GCE	CV	470.4	0–1000	10	This work
Co_3_O_4_ porous NSs/GCE	DPV	3566.5	0–800	12	This work

Abbreviations: Co_3_O_4_, cobalt oxide; GCE, glassy carbon electrode; CV, cyclic voltammetry; Ag, silver; Fe_2_O_3_, iron oxide; PANI, polyaniline; DPV, differential pulse voltammetry; ZnO, zinc oxide; Au, gold; CVD, chemical vapour deposition; Cu_2_O, cuprous oxide; SiO_2_, silicon dioxide; MWCNT, multiwalled carbon nanotubes; SWV, square-wave voltammetry; PPD, p-phenylenediamine; PrB, Prussian blue; SPCE, screen-printed-carbon-electrode; ITO, indium-doped tin oxide; rGO, reduced graphene oxide; NPs, nanoparticles; LSV, linear-sweep voltammetry, ZIF-11, zeolite imidazole framework; g-C_3_N_4_, graphitic-like carbon nitride; NSs, nanosheets.

**Table 2:** Uric acid detection in human serum with cobalt oxide/GCE sensor.

Sample	Added Uric Acid (µM)	Found (µM)	Recovery (%)	RSD (%) (n = 3)
Human serum	0	282	-	-
	10	291.6	96	2.38
	50	329.8	95.6	2.92
	100	379	97	3.45
	500	771	98.2	4.25

## Data Availability

Not applicable.
